# Supplementary figures and images for: RNAhub - an automated pipeline to search and align RNA homologs with secondary structure assessment
Source: bioRxiv. 2025 Apr 8:2025.03.11.642701. Preprint. [Version 3] doi: 10.1101/2025.03.11.642701 (PMC11952402; doi:10.1101/2025.03.11.642701)

infernai\_1.cacofold

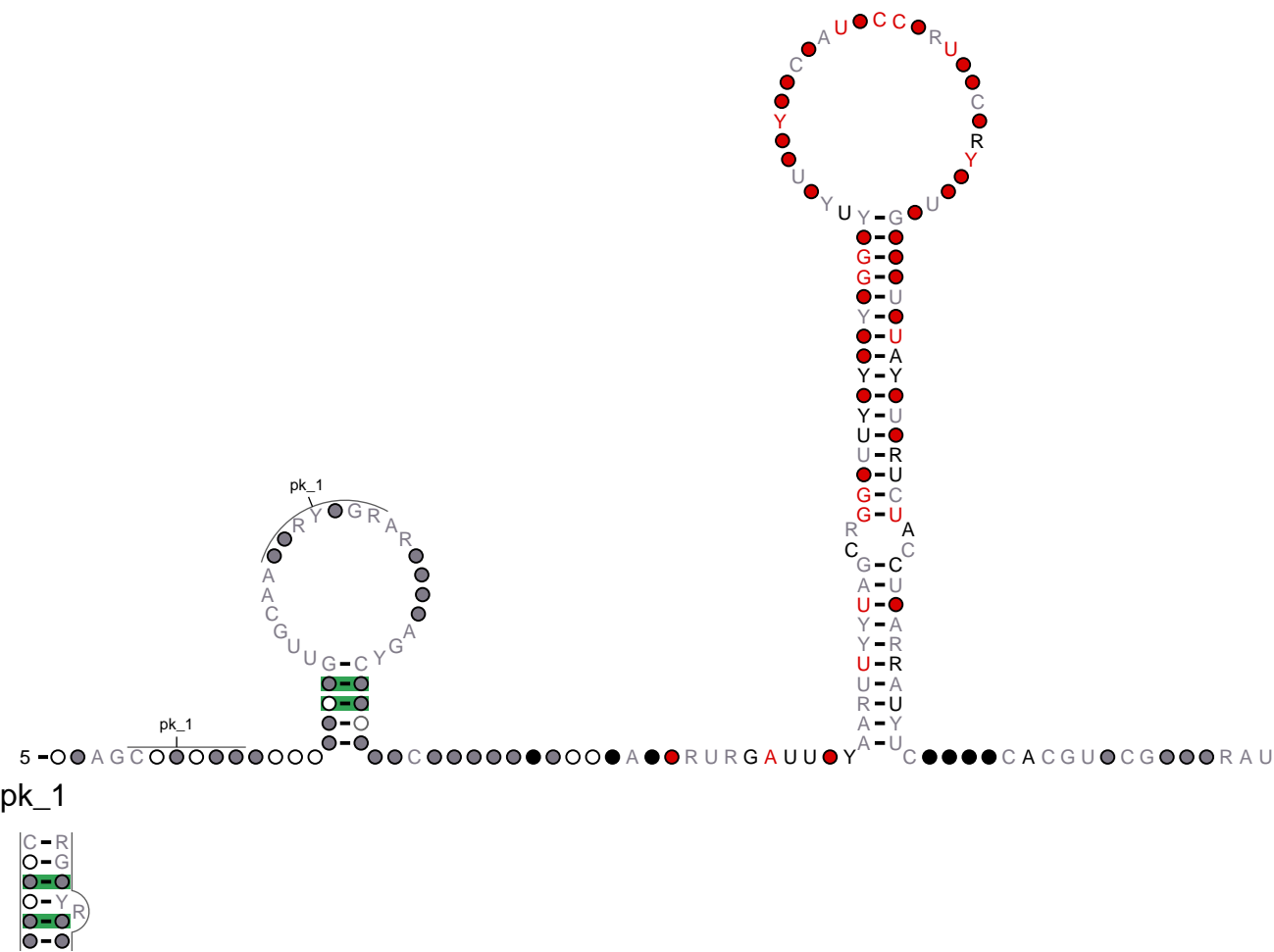

Supplement: Supplement 1 [file media-1.gz › supplemental_material/xrRNA/RNAhub_genomes/rscape_infernal/infernal_1.cacofold.R2R.sto.pdf]

seq+RF04222\_seed\_1

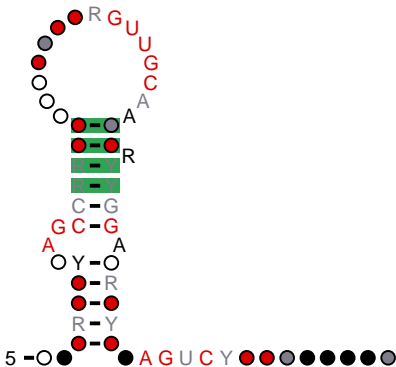

Supplement: Supplement 1 [file media-1.gz › supplemental_material/xrRNA/RNAhub_rfam/rscape_rfam/seq+RF04222_seed_1.R2R.sto.pdf]

# CWC15.cacofold

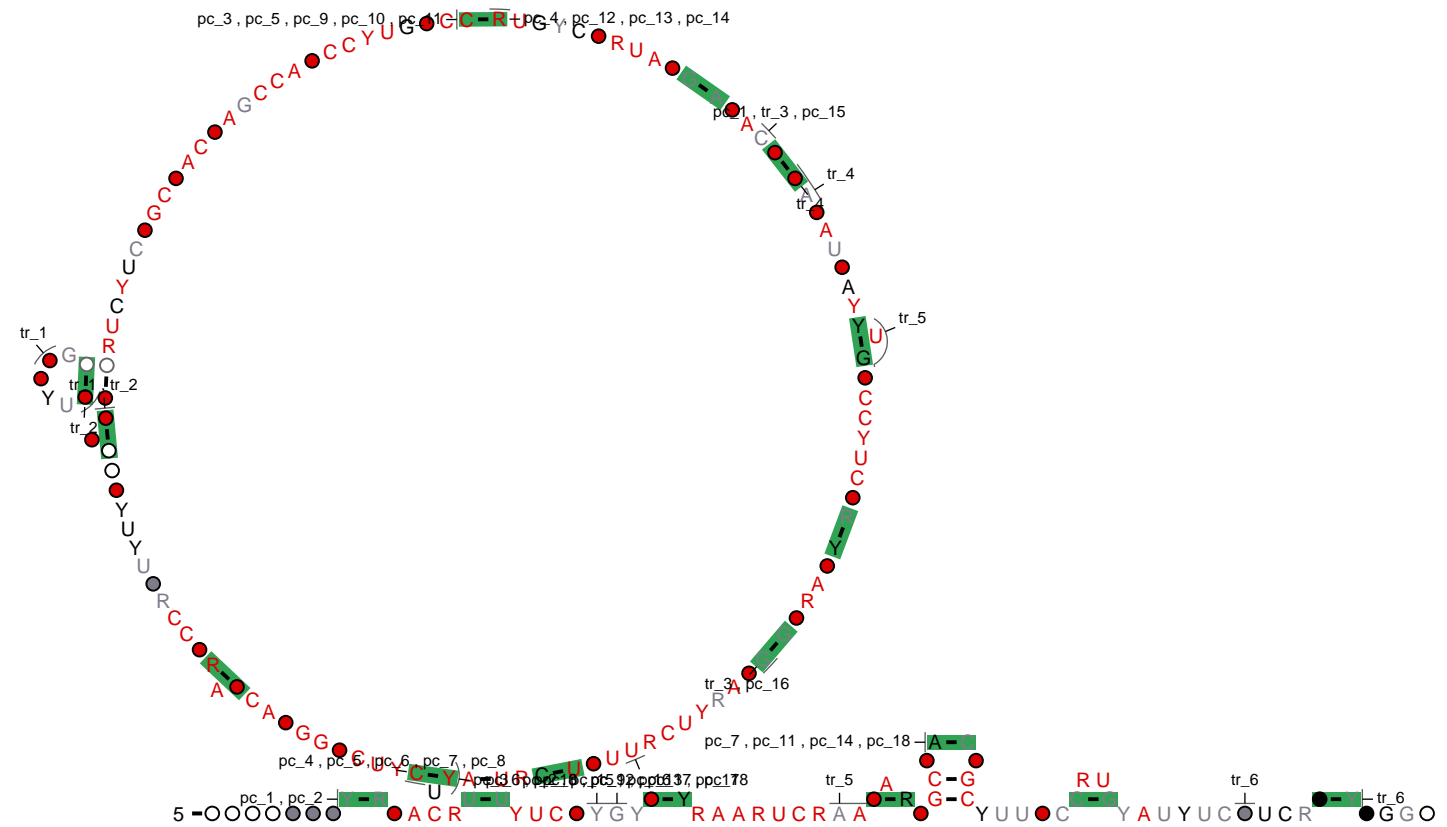

pc\_1 pc\_10 pc\_11 pc\_12

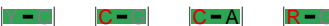

pc\_13

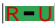

pc\_18

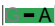

pc\_6

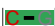

tr\_2

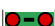

pc\_14 pc\_15 pc\_16 pc\_17

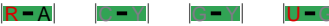

pc\_2 pc\_3 pc\_4 pc\_5

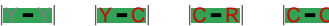

pc\_7 pc\_8 pc\_9 tr\_1

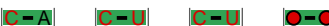

tr\_3 tr\_4 tr\_5 tr\_6

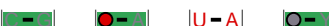

Supplement: Supplement 1 [file media-1.gz › supplemental_material/CWC15_YDR163W/rscape_output/CWC15.cacofold.R2R.sto.pdf]

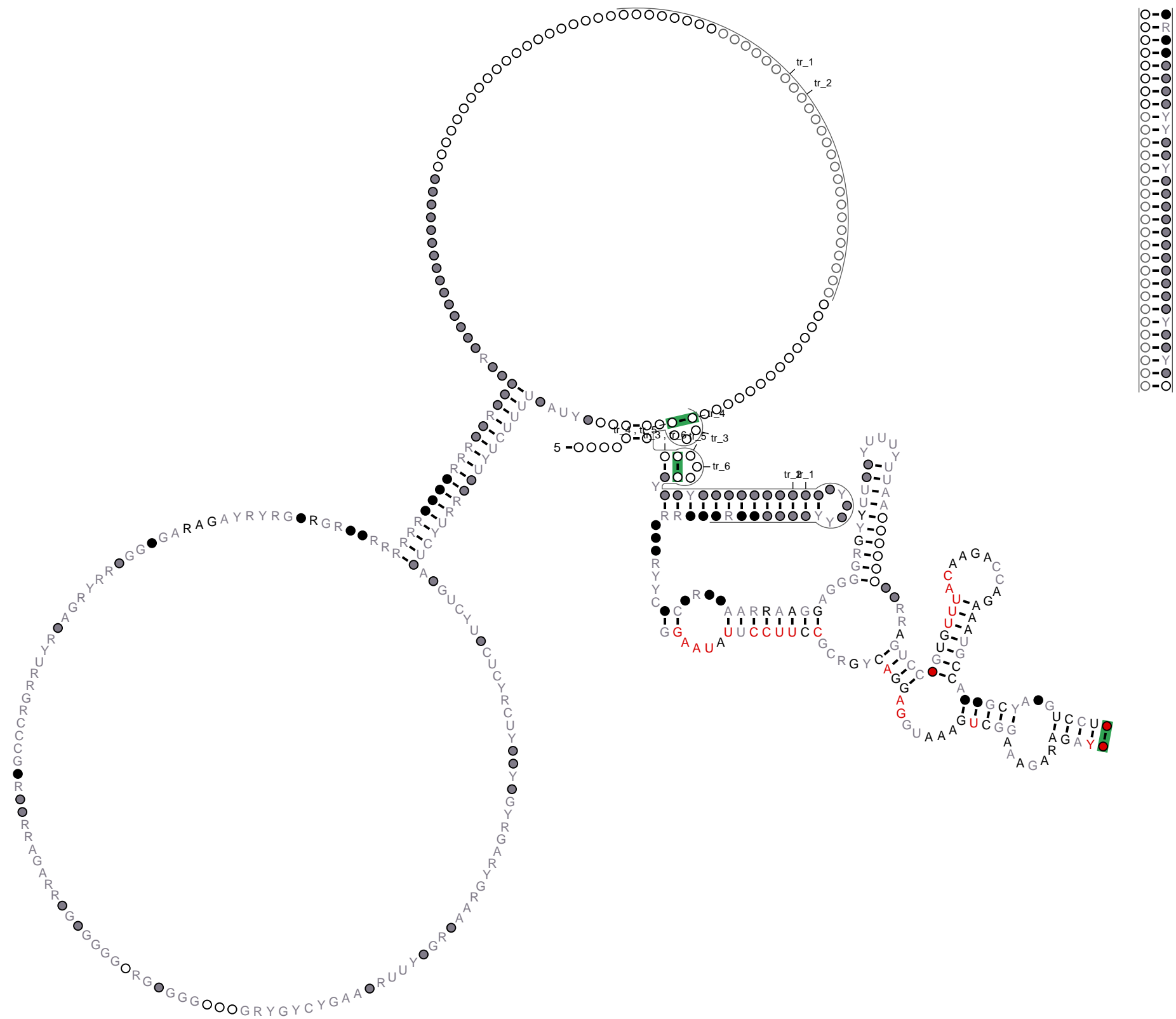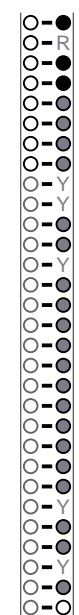

tr\_2

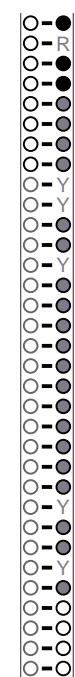

tr\_3

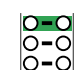

tr\_4

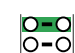

tr\_5

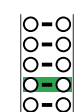

tr\_6

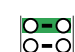

Supplement: Supplement 1 [file media-1.gz › supplemental_material/HOTAIR_D1/rscape_output/HOTAIR_D1_Homo_sapiens_1-526.cacofold.R2R.sto.pdf]

infernai\_1

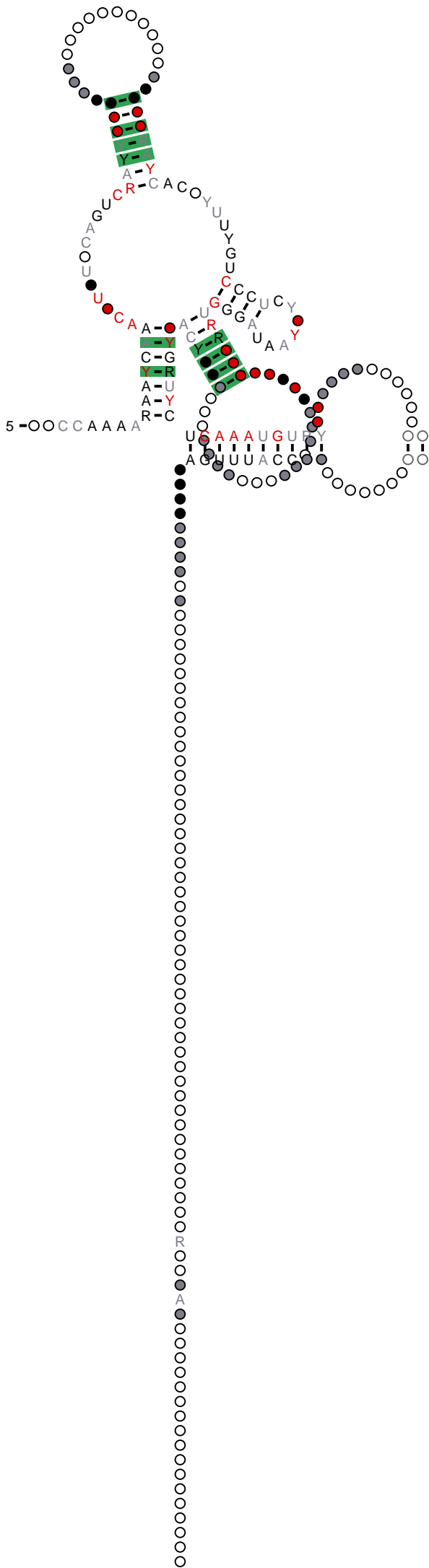

Supplement: Supplement 1 [file media-1.gz › supplemental_material/GLY1/rscape_infernal/infernal_1.R2R.sto.pdf]

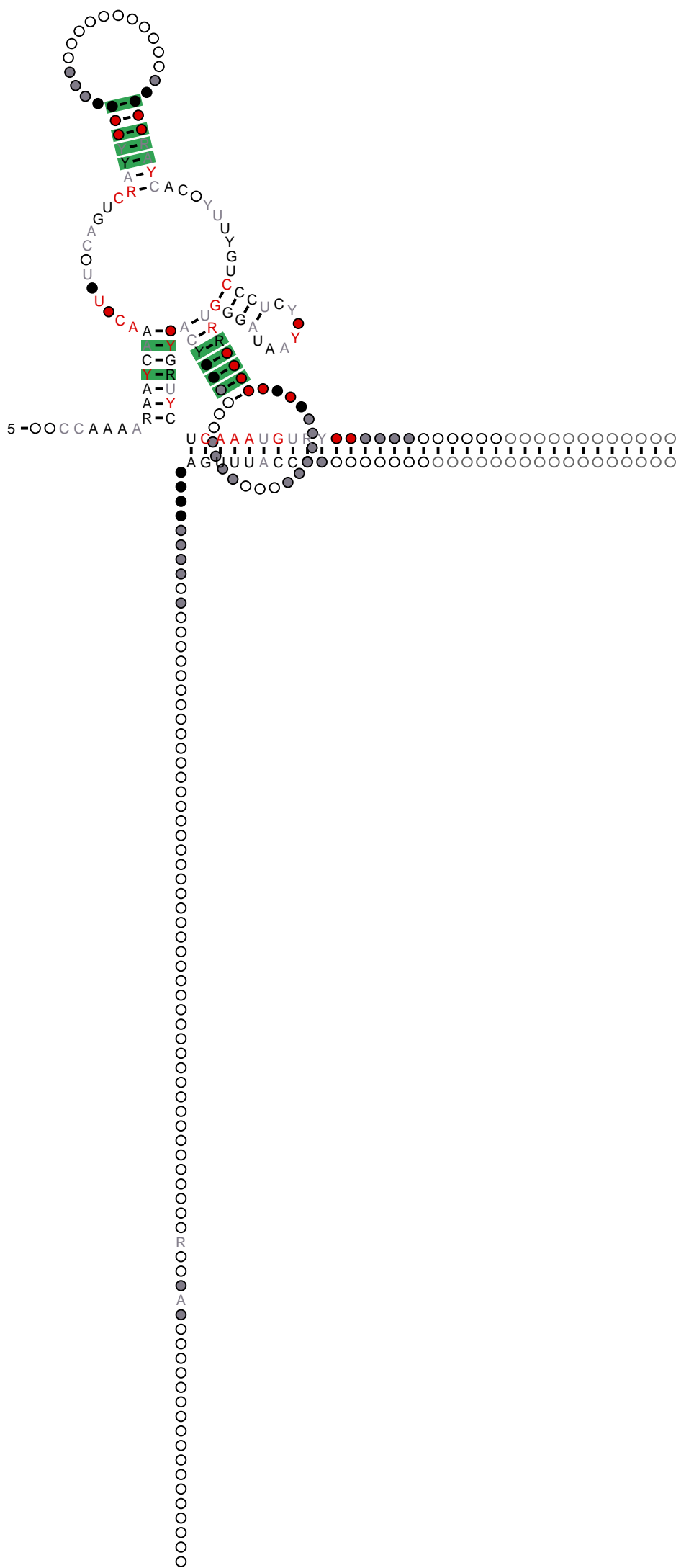

Supplement: Supplement 1 [file media-1.gz › supplemental_material/GLY1/rscape_infernal/infernal_1.cacofold.R2R.sto.pdf]
