# Supplementary material for: RNAhub - an automated pipeline to search and align RNA homologs with secondary structure assessment
Source: bioRxiv. 2025 Apr 8:2025.03.11.642701. Preprint. [Version 3] doi: 10.1101/2025.03.11.642701 (PMC11952402; doi:10.1101/2025.03.11.642701)
Supplement: Supplement 2 [file NIHPP2025.03.11.642701v3-supplement-2.pdf]

bioRxiv preprint doi: https://doi.org/10.1101/2025.03.11.642701; this version posted April 8, 2025. The copyright holder for this preprint (which was not certified by peer review) is the author/funder, who has granted bioRxiv a license to display the preprint in perpetuity. It is made available under aCC-BY 4.0 International license.

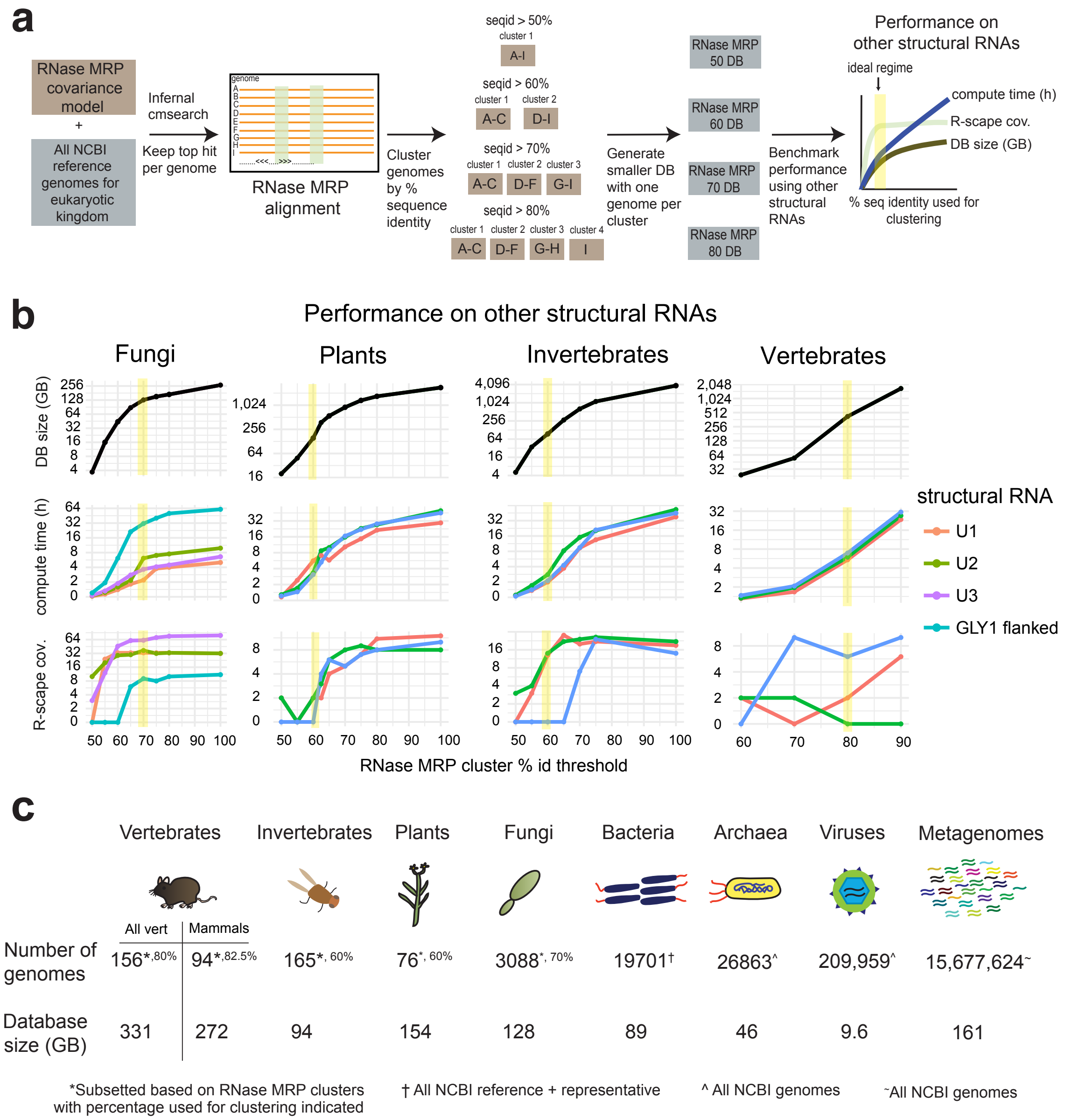

Figure S1: **Clustering of genomes.** (a) To reduce the size of eukaryotic databases, we clustered genomes based on their RNase MRP sequence (a highly conserved structural RNA), and kept one per cluster to construct smaller genome databases. We determined the ideal regime as the subclustered genome database that maximizes the tradeoff between the number of R-scape covariations vs. compute time and database (DB) size. (b) Database size, compute time, and R-scape covariation tradeoff (in log scale) for three non-RNase MRP structural RNAs using clustering thresholds ranging from 0.50 to 1.00, in increments of 0.05. The ideal regime that balances covariation against DB size and compute time is highlighted in yellow (c) Genome databases available in RNAhub, based on either the clustering at the ideal regime for eukaryotic genomes, or all reference NCBI for bacteria, and all NCBI archaea and viral genomes.
