# Supplementary material for: RNAhub - an automated pipeline to search and align RNA homologs with secondary structure assessment
Source: bioRxiv. 2025 Apr 8:2025.03.11.642701. Preprint. [Version 3] doi: 10.1101/2025.03.11.642701 (PMC11952402; doi:10.1101/2025.03.11.642701)

seq+RF04222_seed_1.cacofold

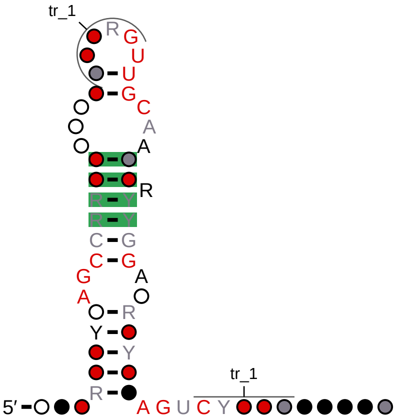

tr_1

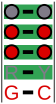

Supplement: Supplement 1 [file seq+RF04222_seed_1.cacofold.R2R.sto.pdf › supplemental_material/xrRNA/RNAhub_rfam/rscape_rfam/seq+RF04222_seed_1.cacofold.R2R.sto.pdf]
